# Dental Notes on the Late Civil War

**Published:** 1868-01

**Authors:** W. Leigh Burton

**Affiliations:** Richmond, Va., Dental Surgeon in the late Confederate Army; Secretary of the Virginia Dental Society.


### ARTICLE III.

### *Dental Notes on the late Civil War.*

By W. Leigh Burton, Richmond, Va.,

Dental Surgeon in the late Confederate Army; Secretary of the Virginia Dental Society.

In contributing this or similar articles to the dental literature of the day, the writer wishes it to be understood at the start, that he is not doing so with the view of keeping up or encouraging any sectional feeling or rancor. Narrow-minded bigots or fanatics may discover evidences of disloyalty or rebellion in them, but his real purpose is to place upon record, facts and incidents connected with dental surgery, precisely as if he had been writing of the English, Austrian or Feejeean army. No one, it is presumed, can be so incredulous as to dispute the fact that, at no very remote period, Confederate armies *did* exist; that they were commanded by officers of fair ability; that they lost and won battles; captured and exchanged prisoners; were provided with hospitals for the treatment of sick and wounded soldiers; and that a good many of them died; and some lived "to fight another day." Nor can it be denied that the failure of the Southern states to attain a separate nationality, was owing mainly to the scarcity of men and the materials of war—these matters having become a part of the history of the times.

The drawbacks caused by the scarcity of men and the materials of war were felt in every department of the Confederate government, but in none more sensibly than that in the medical department, and associated with it, that of dentistry. Medicines and surgical instruments having, at the commencement of hostilities been declared contraband of war by the United States government, it is not to be wondered at that the Confederates stood sorely in need of the most essential remedies in the materia medica. To depend upon running the blockade was but a precarious business at best; and the laboratories established at Charlotte, N. C., and other points, while they were able to supply chloroform, ether, and other medicines of tolerably fair qualities, were totally unable to furnish quinine and other rare and valuable remedies.

It should be enough to refute the charge of inhumanity so often brought against the Confederate authorities to state that the medical department, particularly, used every available means to remedy the evils caused by an inadequate supply of medicines of Northern and foreign manufacture. In the year 1863, Surgeon F. P. Porcher, by order of Surgeon General Moore, prepared at the expense of great labor and research, a book which tended greatly to alleviate the inconveniences arising from an insufficient stock of medicines. The writer has before him a copy of this rare and valuable work. It is entitled, "Resources of the Southern Fields and Forests, Medical, Economical, and Agricultural. Being also a Medical Botany of the Confederate States; with practical information on the useful properties of the trees, plants, and shrubs." Attached to the book is a "Standard Supply Table of the Indigenous Remedies for Field Service and Sick in General Hospital."

The fact of the publication of this book is known to but comparatively few persons at the South, and from its great scarcity, it is doubtful if a copy of it is to be found among the archives of the "rebellion" at Washington city. Almost every conceivable subject indicated by its title is treated of. Not only are full directions given for the preparation of medicines, but the same careful instructions are laid down for making certain kinds of cement or for the management of silk worms.

Concerning the scarcity of men for field service, it was not an extravagant metaphor of General Grant's when, towards the close of the war, he telegraphed to the authorities at Washington that the Confederates were "robbing the cradle and the grave." As bearing upon the need for men and relating also to operations in dentistry, the following circular is published:

War Department,
Surgeon General's Office,
Richmond, Va., *Dec.* 3, 1864.

Circular
*No.* 22.

*To Medical Directors of Hospitals.*

I. Soldiers who have lost the hand or arm and otherwise healthy, but are incompetent to perform clerical duty, can, in the use of a pistol, act as efficient guards for Hospitals and Purveying Depots. The majority of the guard can be composed of such men. Medical Directors of Hospitals will ascertain the number of Dragoon Pistols needed for this purpose in the Hospitals under their charge, and make requisition accordingly upon the Ordnance Department in Richmond.

II. Dental Surgeons are required to make monthly, to this office, to be forwarded by the 5th of the month through the Medical Director, written reports, in which will be noted in separate columns, and separately for each hospital, the number and character of each surgical operation performed during the month.

(Signed,) SAM'L PRESTON MOORE,
*Surgeon General C. S. A.*

It is presumed the "Dragoon Pistols" referred to in paragraph I, must have been *self-cockers*, for had they been of the ordinary army pattern, it is difficult to understand how they could have been made available in the *hand* of a one-armed man.

At the commencement of, and during the war, there was only one firm in the South engaged in the manufacture of gold foil and dentists' materials—Messrs. Brown and Hape of Atlanta, Ga. Owing to the rigid conscription laws passed by Congress, the principals and employees of this establishment were conscribed at an early period, and consequently its operations ceased ; until through the intercession of the surgeon general and the entire dental profession, they were exempted from military services. They had scarcely resumed operations, however, when they were forced to remove to Augusta by the evacuation of Atlanta, by the Confederate forces. But misfortune seemed to cling to these enterprising gentlemen. One of the firm having received a permit to purchase machinery and materials for the manufacturer of gold foil, abroad, was about entering a Southern port, when the ship was wrecked and everything on board went to the bottom of the sea!

It was not only gold foil that became scarce and high priced, but soon after the assignment of dentists to duty in hospitals, the supply of files become entirely exhausted. All efforts to obtain them through the blockade having failed, the medical department determined to try the experiment of their domestic manufacture, and consequently the following requisition was made.

CONFEDERATE STATES OF AMERICA,
Surgeon General's Office,
RICHMOND, VA., *Febr'y* 23, 1865.

GENERAL:

I respectfully request the privilege of procuring from your Department two lbs. of sheet steel, for the purpose of manufacturing files for operations on the teeth. Very respectfully,

BRIG. GEN. GORGAS, Your ob. servant,
Chief of Ordnance, S. P. MOORE,
Richmond. *Surg. Gen'l C. S. A.*

Although a dentist's file had never before been made south of the Potomac, the success of this experiment was complete. It may be true they were not equal to Murphy's in point of finish and appearance, but nevertheless they answered every purpose admirably. The "two lbs. of sheet steel," referred to in the above requisition was required for files for the front teeth. The "knife edge" file had been successfully made before, and after it had been ascertained that every variety of the instrument could be made in Richmond, dentists were kept constantly supplied.

Upon the assignment of a dentist to a hospital, it may be said as a rule that, the surgeon in charge afforded him every facility in his power for his operating to the best advantage. A room with a good light, cold and warm water, soap and towels, and a servant or soldier, were invariably provided. The making of the operating chair was entrusted to the hospital carpenter, and generally constructed by a rude design drawn in pencil. A tin basin placed upon a bench or stool answered for a spittoon. In cold weather a good fire was kept constantly burning in the room—that is to say, when the hospital was supplied with wood.

Dental patients were divided into three classes: those from the front; the convalescent; and the sick. Those from the front were invariably treated first, in order that they might return to their respective commands. They entered the hopital especially for dental operations and were discharged after the completion of them. If it was argued by some officers that men from the front made the requirement of dental operations a pretext for absence from their commands, in order to have a day's relaxation in the city, they generally paid for it by the loss of one or more teeth. And even if they relished the operation, there was but little opportunity afforded them for any other enjoyment. After being discharged from the hospital they were conveyed, under guard, to the "Soldier's Home," where they remained until the "Provost Guard" escorted them to their commands. These measures appear harsh, and while no doubt they were very necessary in some cases, many men were deterred from having the benefit of dental operations rather than submit to them.

Officers or men not registered in hospitals were not entitled to dental operations unless upon a special order of the Medical Director of the department, and they were never granted except in urgent cases. The form is here given :

MEDICAL DIRECTOR'S OFFICE,
*Richmond*,..............1864.

Surgeon Dentist..............................................................will admit
................................................for attention to teeth.

By order of Medical Director.

(Signed,) FRANS. D. CUNNINGHAM,
*Surgeon.*

Dentists were provided with ambulances to enable them to reach the hospitals as conveniently and with as little fatigue as possible. Many of the hospitals being at a considerable distance from the city, and the dentist having to carry his instruments and materials, this arrangement was eminently proper, as it is well known that it is impossible to perform any delicate operation after having carried in the hand for two miles or more, a parcel weighing several pounds. But dentists were not allowed this privilege without an effort having been made in certain quarters to break up the arrangement. Some of the assistant surgeons having to walk to their posts of duty, became jealous of the privilege allowed dentists, and not only complained of it, but tried in various ways to have it discontinued. In a case of the writer's, the ambulance having failed to call for him at the usual hour, and being compelled to walk to the hospital, he was of course behind time. This apparent dereliction of duty was reported promptly to the surgeon in charge, and eventually went the rounds of all official papers until it became at last so filled with endorsations that it was positively frightful to behold. The point made against the offender was, that as many of the surgeons were in the daily habit of *walking* to the hospital, the non-arrival of the ambulance could not be received as an excuse for not being in place at the proper time. It is enough to say, however, that this little scheme failed most signally, and that dentists had the use of ambulances up to the last days of the Confederacy.

It is pleasant to turn from these little weaknesses of poor human nature to the contemplation of a noble and magnanimous character, such as the late F. W. Hancock, surgeon in charge of Jackson Hospital, possessed. It would be no fulsome flattery to his memory to say that he was a conscientious officer, a good man, and a gentleman in its fullest meaning. Many are still living to bless the memory of him, who while they were stretched upon a wretched hospital bed, racked with fever and pain, cut off from intercourse with home and friends, and dispirited by military reverses, have been cheered and comforted by his kind words and approving smile. Many, alas! who had tearfully looked into his kind face for some signs of a hope of life, went before him, and those acres of little hillocks near Holywood, attest to the fearful ravages of the grim monster!

Constantly besieged by executive business, and often being harrassed at not being able to obtain supplies, Surgeon Hancock, still found the time to give his personal attention to the minutest details of his immense hospital, having the capacity for the accommodation of ten thousand patients. In no department of his hospital did he take a livelier interest than that of Dental Surgery. He was fully alive to the importance of it as effecting the health and comfort of the men under his charge, and nothing was left undone by him which could possibly aid in its application to all who desired it. From the connection of the writer with some of the largest hospitals in the late Confederate States, it appears to him that before closing this article, he should have something to say upon a subject, about which, much apparent ignorance prevails—the supply of food.

Of course it would be quixotic in any writer to attempt to beat down the prejudices produced by the alleged barbarities practiced at Andersonville and the Libby. Still the truth may be told for all that ; and if it should be the means of changing to any extent the opinions of even a very small number of people, some good, at any rate, will have been accomplished. The writer states on his own knowledge, that at times, he has known the employees and attachees of hospitals to subsist for three weeks solely upon *corn bread and sorghum !* Think of matrons, ladies, who during other and better days had been accustomed to every luxury, living upon such miserable diet ! Of course there were times when the fare was better, but it frequently happened that all lines of railway or the canal would be cut by a raiding party, when great scarcity of food would ensue. And how did the poor Confederate in the field fare? On canned meats and fruits, sardines, bologna sausage, and wholesome bread? Alas, no ! Occasionally he may have revelled in the luxury of fresh meat and wheat bread, but more frequently—particularly during the last days of the Confederacy, his fare consisted only of corn bread washed down by "Confederate coffee," or else he had to be, and was satisfied with, roasting ears or parched corn.

Many more facts might be adduced in proof of the great scarcity of food existing in the Southern States during the four terrible years of war and carnage; but enough has been said in this article to prove that this scarcity did not extend to food alone. With the ports of the world closed against them, with a currency depreciating every hour from the most shameful mismanagement; and having to contend against such enormous odds, the great wonder is, how even a noble and heroic people, believing honestly in the justness of their cause, could hold out so long.

